# “We’re Just Sitting Ducks”: Recurrent Household Flooding as An Underreported Environmental Health Threat in Detroit’s Changing Climate

**DOI:** 10.3390/ijerph16010006

**Published:** 2018-12-20

**Authors:** Natalie R. Sampson, Carmel E. Price, Julia Kassem, Jessica Doan, Janine Hussein

**Affiliations:** 1 College of Education, Health, & Human Services, 19000 Hubbard Dr., Dearborn, MI 48126, USA; jhdoan@umich.edu (J.D.); jhussein@umich.edu (J.H.); 2 College of Arts, Sciences, & Letters, 4901 Evergreen Rd, Dearborn, MI 48128, USA; carmelp@umich.edu (C.E.P.); jkassem1996@gmail.com (J.K.)

**Keywords:** flooding, water, infrastructure, climate change, vulnerability, risk communication, disinvestment

## Abstract

Recurrent inland urban flooding is an understudied phenomenon that warrants greater attention, particularly in post-industrial cities where aging infrastructure, disinvestment, and climate change threaten public health. We conducted semi-structured interviews in 2017–2018 with 18 Detroit residents experiencing recurrent household flooding. We used standard qualitative coding analysis to generate 30 theoretically- and in vivo- derived themes related to flood experience, socioeconomic and health factors, and household, community, and policy interventions for reducing environmental exposures before, during, and after flood events. Snowball sampling yielded interviewees across both high- and low-risk areas for flood events, indicating vulnerability may be widespread and undocumented in formal ways. Residents described exposure to diverse risk factors for chronic and infectious diseases, particularly for seniors and young children, and emphasized stressors associated with repeated economic loss and uncertainty. Opinions varied on the adequacy, responsibility, and equity of local and federal relief funding and programs. We expand knowledge of flood-related vulnerability, offer innovative suggestions for risk communication based on residents’ experiences, and recommend additional research for documenting patterns of recurrent flooding and response, even for precipitation events that are not characterized as extreme or disaster-level in the media or by agencies. These findings should guide local public health, emergency preparedness, sustainability, water and sewage, and community leaders in post-industrial cities.

## 1. Introduction

For millennia, humans have concerned themselves with droughts and floods and managed stormwater to protect public health in changing environments [1,2]. Ancient Greek and Roman infrastructure provide evidence of ornate aqueducts and downspouts to transport clean water and sanitary systems that separated sewage and burial sites from drinking water [3]. Today, increasingly complex systems are used to manage water with growing demands in highly populated urban areas where aging infrastructure and climate change threaten health. For example, according to the U.S. Environmental Protection Agency [4], in the U.S., about 860 communities have combined sewage systems serving 40 million people in which a single pipe collects and transports stormwater and sewage. This means that during peak flows, human and industrial wastes may put the system over capacity, which leads to pollutants in local waterbodies. As climate change alters our natural water cycle [5], our infrastructure must evolve to ensure public health.

Flooding accounts for 75% of all Presidential Disaster Declarations in the U.S. [6]. In simple terms, “the most common cause of flooding is water due to rain and/or snowmelt that accumulates faster than soils can absorb it or rivers can carry it away [6].” Nearly all communities are susceptible on some level, and few communities are fully prepared or protected. In the context of climate change, much attention has been given to coastal surge flooding and tropical storms that will have devastating effects costing billions. In the U.S., we have already seen a rise of sea level in coastal areas by 7 to 8 inches, on average, since 1900, and expect as much as 1.2 feet by 2050 and 4.3 feet by 2100 [7]. However, inland flooding also warrants significant attention, particularly given an upward trend [8]. Further, in inland urban communities, the relatively high amount of paved impermeable surfaces hinders natural stormwater management, which contributes to excess run-off and regular neighborhood or household flooding that may occur even during less than extreme events [9]. In 2016, inland flood events—both fluvial (i.e., riverine flooding) and pluvial (i.e., surface flooding)—cost the U.S. 4 billion dollars, which was double any previous records since tracking started in 1980 [10]. Further, as Howell and Elliot [11] note, increase in “two defining social problems of our day—wealth inequality and rising natural hazard damages—are dynamically linked.” An understanding of who is vulnerable and how funding is spent on flood prevention, as well as insurance, assistance, or relief must be pursued to reduce threats to environmental health equity.

Threats to public health manifest in multiple ways during and after flood events, which can vary based on range in intensity and type (e.g., surge, fluvial, pluvial) of flood, and many factors increase one’s vulnerability related to morbidity and mortality [12]. Documented health effects from floods may include injury, carbon monoxide poisoning, psychological distress, respiratory illness, and gastrointestinal illnesses, among others [13,14,15,16]. Some outcomes are acute and occur during or immediately after, whereas other outcomes may be chronic or long term, such as psychological effects for those that are temporarily or permanently displaced or respiratory health effects from mold exposure for those unable to afford proper post-flood removal of water or sewage in households [16]. Lowe et al. [12] conducted a systematic review and learned that during floods, risks for psychological and physical health effects are higher among females, seniors, and children. Additionally, young men (ages 10–29 years) may be most as risk for mortality during and after flood events compared to the general population [12]. They concluded that multiple other risk factors adversely affect health outcomes: greater flood depth or flood trauma, existing illnesses, medication interruption, low education or socio-economic status, and trauma from previous flood experiences. Additionally, among communities at risk for natural disasters including flood events, tangible and emotional social support are potential protective factors to consider when designing preventative and response strategies wherein family, friends, or neighbors may affect one’s behavioral responses to emergency weather events [16,17,18]. In contrast, however, some research suggests that high levels of social support may actually reduce people’s sense of their own vulnerability to health effects in extreme weather events [19,20,21,22]. Qualitative research offers needed perspectives on risk and protective factors before, during, and after flood events, or what has been referred to as precaution, coping, and recovery [23].

Flood risk management is often technocratic, without integration of theoretically- and evidence-based risk communication strategies that consider public perceptions [24] and multiple dimensions of vulnerability. Although a body of evidence has emerged on best practices [25], social science has been slow in offering theoretical frameworks to fully understand risk perception and its health implications related to flood events [26,27]. Drawing on extensive quantitative and qualitative research across several European nations, O’Sullivan and colleagues [28] suggest that knowledge of one’s risk level is necessary but not sufficient in motivating residents’ personal flood risk management efforts. For example, flood risk maps and probability of flood events in nearby waterbodies are often publicly available online, but this information is not often accessed, understandable, in plain language, or easily translated into household risk preparedness [29]. Further, few flood-related risk communication studies examine predictive factors that may determine one’s adaptive capacity in recurrent non-disaster-level flood events.

### 1.1. Aging Infrastructure, Extreme Precipitation & Health in Detroit, MI

Detroit, MI and countless U.S. cities are grappling with how to address aging water infrastructure, which has major implications for water access and quality and, thus, public health. As Hanna-Attisha and colleagues [30] explained of the Flint Water Crisis—a statement that parallels many social and economic processes shaping infrastructure issues in other post-industrial cities:

Greater Flint is a postindustrial region of nearly 500,000 people struggling from years of disinvestment by the automobile industry and associated manufacturing activities: the region has lost 77% of its manufacturing employment and 41% of employment overall since 1980 … Greater Flint’s struggles have been amplified by a history of racial discrimination, whereby exclusionary housing practices were common. Such attitudes toward integration later precipitated White flight and emboldened home-rule governance, causing a massive decline in tax revenue for the city. The declining industrial and residential tax bases strained the city’s ability to provide basic services and reversed the public health fortunes of the city and suburbs.

In Detroit, the population dropped by approximately 63% from its peak of 1.8 million residents in 1950 to today’s population of just under 675,000 residents with similar adverse consequences for its tax base and ability to adequately service residents across 139 square miles [31]. Like Detroit, many post-industrial cities’ stormwater and wastewater treatment systems were designed over a century ago with a footprint and technologies that are no longer sustainable to meet the needs of declining populations. Water often becomes less affordable for remaining residents that are economically vulnerable, resulting in major access issues, including frequent shutoffs [32]. And, the costs of operations, upgrading technology, and maintenance to ensure high water quality may feel insurmountable for municipalities, which range in their governance approaches for water management from publicly or privately run to public–private partnerships, at a local or regional level [33].

Likely as a consequence of these infrastructure challenges, household flooding in Detroit may be a repeated public health threat for many. In a small cross-sectional study of 164 heads of households in a Northwest Detroit neighborhood between 2014 and 2015, 52% of residents reported household flooding 1–2 times in the last year, with 12% experiencing three or more events [34]. In a recent survey by the Detroit Office of Sustainability, respondents reported flooding at a rate of very often (13%), somewhat often (23%), and occasionally (32%) [35]. Although it is possible this online survey of approximately 1600 residents did not capture the city’s most vulnerable respondents, the results suggest that recurrent flooding is a widespread issue. In addition to extreme events, such as the 2014 flood, recurrent household flooding may be an underreported phenomenon that is particularly overlooked in non-coastal urban cities experiencing pluvial flooding and may worsen with climate change [8].

In the Midwest U.S., the amount of precipitation falling in the 1% heaviest precipitation events has increased by 37% since the mid-twentieth century [36]. Regional climate change models project increasing frequency, duration and severity of precipitation events over the next thirty years [37]. In Detroit, aging infrastructure includes combined sewage overflow systems, which frequently discharge untreated sewage into the Rouge River and Detroit River. Many low-income neighborhoods in Detroit are at particularly high risk for flooding and sewage back-up into their homes due to the large amount of impervious surface and older housing stock with basements [38]. On 11 August 2014, Metro Detroit experienced record-breaking rainfall—more than 6” in 4 h—which resulted in a federal disaster declaration. This included over 73,000 household claims to Federal Emergency Management Agency (FEMA) for recovery funds in a tri-county region totaling $157 million dollars in approved spending [39]. In Detroit, specifically, 103 residential and non-residential claims for flooding were made in 2014 with 85 paid out by FEMA, totaling $719,514 [40]. Further, in 2018, a class action settlement was approved in which the City of Detroit and the Great Lakes Water Authority were responsible for the creation of a settlement fund totaling $1,300,000 and a Water Bill Credit Fund of $250,000 in response to claims from residents that sewage back-up in their homes in July and August of 2016 were preventable and caused by failed infrastructure [41].

### 1.2. Study Purpose

Practitioners and scholars have largely overlooked recurrent inland urban flooding as a threat to public health. The experiences of those that are particularly vulnerable are often underacknowledged, and these populations are often untapped sources of knowledge vital to informing effective prevention and responses strategies. In this paper, we share excerpts from narratives of those experiencing recurrent household flooding in Detroit, MI in the context of aging infrastructure, disinvestment, and climate change as an urgent threat [42]. From 18 semi-structured interviews with residents, guided by an inductive grounded theory approach [43,44,45], we describe how Detroit residents experience and adapt to recurrent flooding associated with precipitation events—many that may not be characterized as extreme or disaster-level in the media or by agencies.

## 2. Materials and Methods

### 2.1. Data Collection

In 2017, we conducted 18 qualitative in-depth interviews with residents experiencing repeated household flooding throughout Detroit. We achieved theoretical saturation [46] in that key themes began to emerge repeatedly across interviews, signaling that this was an adequate sample size to begin to understand how Detroit residents experience recurrent household flooding and to generate new knowledge about how their experiences relate to existing practices in public health, emergency preparedness, and related fields. We asked community leaders doing work on stormwater management, green infrastructure, water access, and climate change to share our study information with residents that they knew had experienced severe household flooding during the 2014 event and in repeated instances since. We defined ‘flood event’ as an occurrence that entailed basement or household flooding and was considered severe according to respondents. We interviewed heads of household, all over 18 years of age, and relied snowball sampling, as residents and community partners continued to refer others that fit our eligibility criteria. The Institutional Review Board of the University of Michigan approved the study protocol for all interviews (No. IRB00000247), including participant compensation of $25 at the completion of the approximately 40-minute interviews. The semi-structured interview protocol included 16 questions related to participants’ experiences with flooding, particularly regarding their flood-related concerns for finances, health, and their neighbors or loved ones, experience with any community or government resources and assistance sought, and any advice they would give to decision-makers. Interview questions are available upon request. 

### 2.2. Data Analysis

Interviews were audio-recorded and transcribed. Applying grounded theory approaches rather than a priori approaches, we interrogated the data through an iterative process of open and focused coding to identify themes [43,44,45]. First, the five-member research team generated a list of codes by all reading the same three transcripts representing participants from different city districts and varied demographics, given that flooding experiences may vary by neighborhood with regard to one’s vulnerability. After open coding [45], we consolidated and edited themes until an agreed upon list of 30 codes and 16 sub-codes was created (Table 1). Themes included participants’ descriptions of recurrent flooding, health concerns related to their experience with recurrent flooding, and their perspectives on the role of household, community, and government response in addressing these concerns. Many codes aligned with the interview protocol closely. For example, participants were asked about the economic impacts of flooding, and ‘economic impact’ was a distinct code for analysis. Many more codes and subcodes emerged from the data in vivo. For instance, when asked to describe health impacts, in general, participants focused on a variety of topics that became subcodes, including mold, sewage, and respiratory health, as well as vulnerable populations. As another example, when asked to describe their flood experience in general terms, a code emerged for the typology of quantity and severity of flooding. Using Dedoose data management software, we then conducted thematic analyses in which two researchers focus-coded [43] each transcript using the agreed upon codelist. Below, participants are identified with their corresponding city district for context.

## 3. Results

Our results document residents’ perspectives on how recurrent flooding is a public health issue, as well as existing and potential preventative and response strategies needed to protect residents, including recommendations for risk communication before, during, and after flood events. After describing participants (Table 2) and their flood experiences in their own words, we delineate health issues (Table 3) and vulnerable populations as they have described them. As a common theme, we summarize key economic and social stressors (Table 4) experienced by participants, and conclude with their recommendations for households, communities, and decision-makers (Table 5). Table 2, Table 3, Table 4 and Table 5 are provided to give readers sample quotes for major themes and in-text quotes provide additional context for understanding experience and vulnerability, as well as longer, extreme or poignant perspectives. 

### 3.1. Study Participants

**Figure 1:**
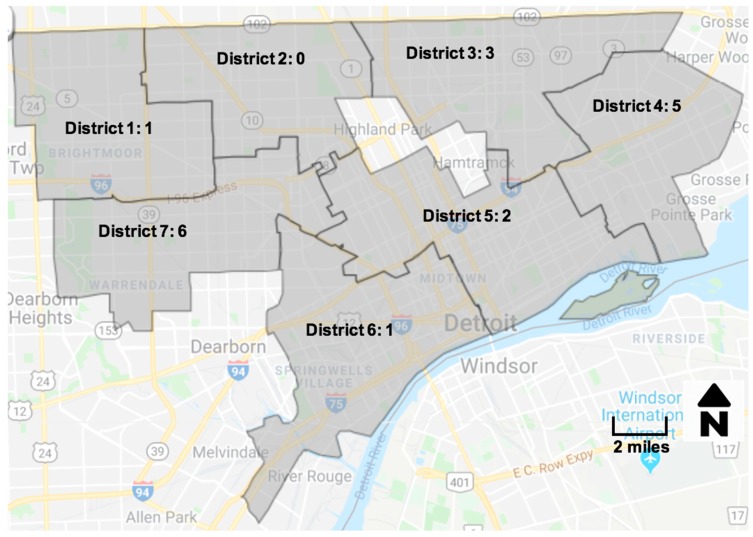
Number of semi-structured interview participants by city district.

We found that snowball sampling yielded participants across the city in several neighborhoods, including neighborhoods not located in flood zones declared high risk by scholars and agencies such as FEMA [48,49]. This indicates that vulnerability may be widespread and undocumented in formal ways. Figure 1 shows the distribution of participants by City of Detroit council districts. On average, participants were 47 years old and 50% identified as female (Table 2). By race and ethnicity, participants self-identified as Black/African American (13), Arab American (2), Hispanic (2), and White (2). Approximately 83% of participants owned their homes. On average, participants reported living in Detroit for 47 years and in their current home for 25 years. Participants reported that they had experienced a range of 3–10 flood events (mean = 4.2) in their household since 2014, ranging from 3 to “every time it rains heavy” (selecting ‘10’ when asked to quantify).

While qualitative research is typically intended to provide rich descriptions rather than a representative sample, it may be helpful to know how the study population compares to that of Detroit, MI, USA [50]. Median age in Detroit is 34.8. With regards to race and ethnicity, the city is 82.7 Black and 10.6% White, while 6.8% of the population identifies as Hispanic and no reliable data exists to understand the size of the Arab American, Middle Eastern, or North African population. In Detroit, 51.1% of occupied households are occupied by owners rather than renters. According to the 2016 American Community Survey, 43.0% of the population have lived in their home since 2010 or later. 

### 3.2. Experiences with & Impacts of Flooding

In general, participants described flooding as a traumatic and unexpected experience. Several explained their metrics for gauging severity by assessing, “rain water or sewage,” “drain or foundation” and “what step,”—meaning, to what step in their basement did the flood water reach? Participants told their stories of household flooding from 6 inches to four feet in their basements to one case of nearly filling their basements and reaching the main floor. They also expressed the recurrent nature, as a man in District 3 explained, “What happened the second time, mine was empty, and I watched it flood like a bathtub,” and “I know it’s gonna happen again ... I know everybody else is like ‘Well this is just once in a lifetime.’ After six times, I’m a believer.” Most came to expect and/or fear additional flooding during heavy precipitation events.

Participants described several ways the flooding affected their own health or the health of neighbors, friends, or family. There were countless examples from participants elaborating on these concerns with sample statements in Table 3. They described exposures that concerned them, including infectious diseases, mold, and sewage, as well as specific health outcomes related to respiratory health, safety, mental health, and quality of life. This frequently included stories of how they or loved ones dealt with recurrent mold and sewage exposure, as well as references to repeated adverse experiences impacting quality of life—from displacement to increased anxiety during regular rain events, from loss of personal items with sentimental value to managing costs of damage and clean up.

Aligning with existing literature [12,13,14,15,16,17,18], participants also described ways that specific populations were vulnerable to these effects, particularly those with existing health conditions or disabilities and seniors or children. Some expressed a need for additional community or government support for these vulnerable populations both during flood events and afterwards. With regards to response during flood events, a woman in District 7 explained,

I just think I’m more concerned about the elderly and disability … I do have a few family members that are disabled. It’s hard for my mom to get around. So I’m just concerned about, like I said, how can emergency crews, fire departments, and police officers respond to when we’re already lacking in that, that area. How can they get there quickly?

Additionally, immediately following flood events, some participants expressed concerns about individuals, primarily children, not understanding potential exposures, as they were “stomping around in the puddles outside that were raw sewage,” explained a participant in District 3. Several participants described a need for rapid clean up support in households where people, often seniors living alone on fixed incomes, may start cleaning up themselves without proper protection to reduce exposure to infectious disease.

Recurrent flooding may mean long-term exposures for residents if sewage or water damage is not fully cleaned up. A senior woman in District 4 explained her own circumstance,

And I still do have some black mold left. And then I had to find someone to clean up the black mold, and that’s another problem. Who cleans up black mold? So I had to find somebody through all these different programs and the lady said that they’re volunteers. I got in touch with them in 2014. I still haven’t had any help from them.

One participant in District 3 explained, “Y’all haven’t even cleaned the first mess up, and we’re just sitting ducks.” He argued that the city needed to support his senior neighbors in properly cleaning up after the 2014 flood because a few years later they were now experiencing mold in their homes and additional flooding was likely.

Long-term repeated economic loss and potential for displacement, in particular, are chronic stressors that may affect well-being according to participants experiencing recurrent flooding. Participants described loss of valuable irreplaceable personal items, cost of assessment and clean up, damage to appliances, cost of preventative strategies, and even loss of housing altogether (Table 4). Participant descriptions of this loss often included discussion of emotional or mental health impacts. Further, when looking to repair damage or replace items, some participants did not have homeowner’s or renter’s insurance, while others were denied insurance claims or were afraid to file in fear of rates rising or loss of coverage.

Among the 18 participants, nine sought and received FEMA funds, four sought and were denied FEMA funds, and four did not pursue a FEMA claim following Detroit’s 2014 flood disaster. Among those that received funds, several said things like, “I mean, FEMA helped, but not even close to what we lost,” (District 7) and “By the time they got down here they said, you know, that they would compensate you for your cleaning, and they gave me like 264 dollars,” (District 4) which was a small fraction of their costs. A senior man in District 7 explained that he received more than he expected, “But it has to be more than a third [of economic loss] I would think. And they acted more timely than the city ever has.” Those that were denied explained that they “waited too long [to file]” (District 7) or did not properly document their damages (District 3). As one senior woman in District 7 explained, “And my husband said it was because I cleaned up everything real well, that they couldn’t tell, and I didn’t get anything.” A few filed appeals and were repeatedly denied. Lastly, those that did not apply for FEMA funds had multiple and varied reasons: (1) not knowing they could, (2) not “taking a resource away from somebody that may be worse off than I” (District 5), (3) because they “help homeowners but not home renters” (District 6), (4) “It wasn’t a state emergency” (District 5), and (5) “I don’t buy into the FEMA plug. We should be able to provide our own relief because we know we have a problem” (District 3). Opinions on the adequacy and role of local and federal relief funding varied.

Known to impact health in complex ways during or after extreme weather events [17,18,19,20,21,22], social support emerged as a theme across most interviews. As one young woman in District 3 expressed, residents may need emotional social support:

I got this feeling of ‘Okay, it happened. We’re sorry. This is why it happened. Okay, fill out this paperwork and go about your day.’ Until a person lives there and knows what it is like to smell raw sewage or knows what it’s like to slip and fall and break a bone and be lying in raw sewage, you can kind of disconnect from those stories and be like okay, just do this and get your money back. But it’s much more than just money that’s needed to mitigate the situation. It’s just, what are you doing to prevent it? Because you can’t pay me for the stress I feel every time I see a heavy rain happen. There’s no paying for that.

Several participants relied on loved ones for tangible support, specifically a place to stay immediately following a flood event. Some relied on loved ones to help with clean up or provide a place to store items or do mass amounts of post-flooding laundry, but some also mentioned not wanting their friends or family exposed to sewage, as described by a senior woman in District 7, “Everyone wanted to help. We said you’re not coming to help ... you’re not coming to help! My son’s girlfriend has asthma, breathing issues so we said you’re definitely not coming to help.” Again, participants expressed concern for those that are socially isolated, as described by a senior man in District 6, “I am fortunate enough to not be subject to it as bad as other people, but I have people that can’t go anywhere...can’t leave their house .... I am the fortunate one because I have somewhere else to go to.” Nearly all participants discussed some sort of tangible social support they sought or needed.

### 3.3. Strategies for Protecting Residents before, during and after Flood Events

Participants described a variety of household strategies they use to try to prevent household flooding or prevent exposure to potential related health threats. Three of the 18 participants described installing a sump pump, which are devices typically installed in basements to extract excess water and pump it outside of the home to a place that will be less problematic. Others have taken additional strategies to try to protect their homes, including making “a kind of like a cement wall around the house so every time the streets flooded it didn’t get into our basement as easily” (District 7) and another participant, “put silicone on top of the concrete” (District 5). With these technology or structural approaches, some expressed skepticism or reluctance that the strategies would actually work, “We don’t know if that’s going to work, but we’ll see” (District 7) and regarding their new sump pump, another young woman explained, “So that’s a big concern, that it’s not a guarantee for us” (District 3). Many discussed wanting such solutions but being unable to afford them or, for renters, finding it unlikely a landlord would install them.

Participants proposed various steps they now take to protect themselves or their belongings at home. Several participants described how they have emptied their basements in anticipation of future flood events or they closely follow weather warnings and advisories, elevating anything they can before a storm and immediately unplugging appliances. The following strategies were offered by a woman in District 4, who had experienced four major household flood events:

I guess, the first thing that comes to mind is making sure that people don’t come in contact with the water and understand to never touch anything with your bare hands. Don’t wear the same shoes you wear in your basement upstairs. That’s a practice I have, like a basement pair of slippers. So, when I go downstairs, I switch out of my upstairs shoes and put on the basement shoes and leave them downstairs, you know? In the event you may not have sanitized to a level that is safe and that restores the level of cleanliness in your basement, you can at least have some of those practices, at least.

And, other things that I think health practitioners might be able to offer is what to do when you’re exposed: What kind of behaviors might you have? Maybe have all that stuff, ‘What to do when your house floods kits,’ and make sure those are dispersed in flood zones. Including that [information] on our monthly water bill, I think some tips and stuff would be helpful.

Similarly, another young woman in District 3 described the need for a ready-made kit with gloves, a facemask, and goggles, whereas many people just start cleaning without protection to get rid of the sewage and related odors as quickly as possible regardless of potential exposures. She also explained, “document, take pictures, write down everything that was in your basement ... I mean, you’re going into emergency mode naturally, but in order to get back the things you’ve lost, you do need to document what’s happened.” These insights may be vital for other residents likely to experience future household flooding.

Participants also described the need for multiple methods of risk communication—how to prevent risks and how to respond to them. They recommended communicating flood-related risks through weather and emergency apps on phones, on the radio and local news for seniors who may not use mobile devices as a news source, and in community spaces, particularly through senior centers, block clubs, faith-based centers, and cultural centers, as well as the local Community Emergency Response Team coordinated through Detroit’s Homeland Security and Emergency Management Office. Some participants described relying heavily on these community resources for information immediately following the 2014 flood. As one middle-aged woman in District 7 explained:

They may say there’s possibly, you know, possible warnings, but nobody can say, ‘Okay, there’s going to be flooding at exactly six o’clock (laughs), make sure they have everything together...Only thing I can say is that just have a plan for it. I didn’t have a plan for it, nobody did. And the next time this does happen, I mean I know there were a lot of families that were affected way worse than I was and they just, you know, don’t really talk about it but my only thing is just have a plan for it, be prepared, help your children and pray.

Many participants recognized a major challenge with risk communication regarding flooding is that, although heavy precipitation may be somewhat predictable, the resulting severity and location of floods may or may not be predictable. Thus, in addition to messaging during and after a flood, risk communication must occur widely and in advance to support development of household emergency plans. 

Participants connected their flood-related concerns to patterns of depopulation, residential vacancy, and disinvestment that Detroit has experienced, and some expressed mistrust in local government. One senior man in District 4 explained he was unaware of efforts to address flooding, “They’re not designing any programs. You don’t hear from the council, the senators—at all—about anything flood-related.” A woman in District 7 blamed a lack of municipal resources for widespread flooding issues:

… but I don’t know how you could get that in Detroit. I mean there’s so much. I mean I’m not knocking my city. I love my city, but there’s so much that we could be doing, so many things that you have to do. It’s impossible to just say now we need this. They just don’t have the resources for it.

And, one woman in District 4 noted the complexity of the city’s economic and infrastructure challenges coupled with the challenges of having many vacant households in her neighborhood, which required additional municipal management:

I think that there should also be some evaluation of some impact of vacant homes. I can think of the times I’ve seen water flowing into some vacant homes—and what impact does that have on the system of the street or streets that have high vacancy and it’s like a couple people living near them who don’t have that water flow necessary to keep those systems working properly.

Management of stormwater relates to management of vacant land and households, as some participants explained how water infrastructure needs to be recalibrated to accommodate their neighborhood’s changing land use and smaller population. Finally, most participants expressed how the cost of flooding added to compounded extra costs they experienced as Detroiters, which they note are relatively higher than other cities, for example, city taxes, water and stormwater drainage fees, and car and housing insurance.

Participants had many additional suggestions for local leaders related to emergency planning, municipal services, outreach and communication, partnerships, prevention and relief programs, stormwater infrastructure, and utilities (Table 5). Specifically, when asked what advice she would give decision-makers, a woman in District 4 provided a passionate response calling for interagency efforts that reflect the experience of Detroiters:

That’s information that can be gleaned from people who have experienced the floods. And I think hearing those stories—at least I would hope—that would compel them to act more quickly and see this as a public health concern, an economic concern. People have to take off work. People have to spend money that they either don’t have or weren’t planning to spend. And it just puts you in a whole mess of things to do and things to consider. So, as they’re creating those programs, I think talking to people who have experienced these floods can give them this insight into how to design their programs so that they can be effective ... so that they can be time-sensitive and relevant. It doesn’t help me to find out about something 30 days after the fact. It needs to happen rapid response to it. And, talk to other agencies that may provide support in other areas, whether that’s within the Metro area or the state and looking to other communities that have experienced floods more frequently.

Again, participants suggested a range of recommended strategies needed at different points in time to better anticipate frequent household flooding, offer rapid response, and build in long-term efforts to support households and communities in relief and prevention.

## 4. Discussion

### 4.1. Lessons Learned

Recurrent inland urban flooding is an understudied issue that warrants greater attention, particularly in the many places where aging infrastructure, disinvestment, and climate change threaten public health. Recurrent household flooding in Detroit appears to be a constant threat that will remain unless interventions are established with improved risk communication. Mostly, these narratives add depth to existing literature on flood-related vulnerability [12,13,14,15,16,17,18] and highlight how recurrent flooding may exacerbate vulnerability through *repetitive* and *ongoing* exposure to environmental exposures (e.g., mold) and social stressors (e.g., anxiety, repeated loss), particularly for those with limited tangible social support in their relief and recovery efforts. Technological and household or community landscape interventions may be preventative for some residents but these narratives remind us that these are cost prohibitive solutions and limited in their ability to fully counter increasing frequency and intensity of precipitation events and major infrastructure challenges in some parts of the city. They may also provide an “illusion of safety known as the levee effect” ([27], p. 1577). The narratives of those that have experienced this repeated adverse event are vital in informing these potential interventions and offer needed perspectives on risk and protective factors before, during, and after surface flood events.

It is well known that improved risk management and communication is needed to reduce future environmental health exposures associated with flooding [51]. Many existing flood-related risk communication messages present residents with plentiful information, but they are relatively ineffective in increasing residents’ perceived susceptibility of health-related outcomes and intention to prepare [27,29]. Our study emphasizes how uncertainty associated with recurrent household flood events, combined with repeated economic and emotional stressors, may greatly reduce one’s capacity to prevent and manage risks at the household level. These residents’ narratives specifically do provide innovative but practical suggestions including the promotion and content of flood preparedness toolkits or simple strategies to reduce exposure such as ‘basement shoes.’ Our research re-emphasizes that flood-related risk communication campaigns must entail targeted messages [28] that fully recognize social determinants of health, especially in under-resourced communities.

Further, federal and local relief efforts may not adequately respond to inland recurrent flooding among those most at risk [52,53]. Our findings distinctively complement recent research that suggests that uneven FEMA distribution may contribute to social inequality [11,54], and we would add that this may also contribute to environmental health inequities based on which populations become ‘sitting ducks’ with increased risk for long term and ongoing adverse exposures. When synthesized, the narratives of participants suggest that some of the most socially and economically vulnerable individuals may not have the informational or tangible support to successfully pursue relief funds due to lack of documentation, lengthy appeals processes, or the inability to cover costs and seek reimbursement. Instead, it seems as though many residents take on sanitation efforts themselves to reduce costs, often without proper protection from environmental exposures or resources to completely eliminate water, sewage, or mold as potential risk factors for various health outcomes. A few participants did not feel as though FEMA funding was appropriate for their own or the City’s relief efforts, particularly after the August 2014 flood, however. Some argue that both insurance programs, including the National Flood Insurance Program, and relief programs have historically encouraged development or redevelopment in at-risk areas [55,56]. The respective responsibility of households and municipal and federal governments in preparing and responding to increased flooding is a contested issue globally [57,58,59] and may be further complicated in the historical context of disinvestment underlying Detroit’s aging infrastructure. 

This was a small qualitative study that cannot characterize the scale of household flooding in Detroit but it can show the potential severity of the experiences for residents and implications for public health. While we think these are generalizable lessons, more so qualitative research is intended to disentangle an understudied experience or phenomenon. In this case, we are aiming to first descriptively understand how Detroit residents experience recurrent household flooding and generate new knowledge about how their experience relates to existing practices in public health, emergency preparedness, and related fields. Snowball sampling does not necessarily reflect true patterns of this issue, and survey research is underway to estimate citywide patterns of basement flooding and related exposures. Future studies may also need to explore the experience of owners and renters separately to assess the role of landlords in protecting public health. Also, future studies are needed to understand who does or does not pursue financial assistance, including FEMA funding, and who is denied assistance, which may offer additional information on the needs of vulnerable populations in extreme events.

### 4.2. Implications for Detroit and Beyond

Given many citywide efforts are underway towards renewal, revitalization, redevelopment, and reuse of vacant land in post-industrial Detroit [60,61,62], decision-makers must consider the narratives of residents in diverse neighborhoods presented here. Continued attention to issues of equity regarding stormwater management are needed across agencies to protect public health. The first citywide Office of Sustainability was established in Detroit in 2017. This office is currently working on community engagement and interagency collaborations to inform the City’s first sustainability plan and priority strategies, with plans to update a heat and flood vulnerability assessment conducted for the City in 2012 [47]. Also, the Detroit Water and Sewerage Department is currently implementing extensive green infrastructure practices to reduce sewage overflow at 17 specific outfalls in the Upper Rouge Tributary watershed in Northwest Detroit [63] as part of an agreement with the EPA. Under this plan, bioretention sites can capture excess stormwater using natural systems and remove burden from the overall combined sewage system, which could result in fewer household floods. In addition to these efforts, various technological interventions, while costly, present opportunities to reduce household flooding, such as smart stormwater systems with sensors to instigate watershed reconfiguration during extreme precipitation events [64]. Meanwhile, the Detroit Health Department initiated a Community Health Assessment in 2018, creating an opportunity for surveillance of climate-related health issues, such as prevalence of household flooding. Across these agency efforts, ongoing interagency dialogue may be helpful in understanding if and how patterns of recurrent household flooding are altered over time. To address inland urban flooding, sustainability, water and sewage, and public health leaders must respectively and collectively consider their roles in stormwater management and risk communication to address health inequities.

These data were collected at a time when water has become a highly politicized topic in Michigan, which is relevant to many geographies grappling with similar issues in the context of climate change. Starting in 2014, the Flint Water Crisis highlights an ongoing case example of misconduct and negligence by state and local government to protect municipal drinking water in Michigan, which resulted in exposure of 1000 s of residents to unsafe drinking water lead levels, as well as exposure to other chemical and infectious diseases [30]. In 2018, the Michigan Department of Environmental Quality approved a permit from Nestlé to extract 576,000 gallons daily from the White Pine Springs well in the Great Lakes Basin, despite 80,945 public comments arguing to deny the request compared to 75 in favor [65]. Recent testing has uncovered the widespread nature of per- and polyfluoroalkyl substances (PFAS) contamination in Michigan causing a statewide response and several conversations across communities concerned about drinking and recreational waterways [66]. In Detroit, more specifically, issues of flooding coincide with a series of water shutoffs as a related public health concern documented by a community-academic effort, We the People [67]. As identified through a Freedom of Information Act by the Detroit News, the Detroit Water and Sewerage Department shut off water in 30,496 households in 2016 [68]. Also, in Detroit, in August 2018, Detroit Public Schools shut off drinking water in all schools after testing showed lead and/or copper levels exceeded safe levels set by the Safe Drinking Water Act in several schools. While household flooding in Detroit has its own risk factors and raises distinct threats to health separate from these other issues, it further illustrates the complexity of managing water access and quality responsibly and equitably in the changing environment of the Great Lakes region—home to 21% of the world’s supply of surface fresh water.

## 5. Conclusions

The U.S. Water Alliance explains that water equity occurs, “when all communities have access to safe, clean, affordable drinking water and wastewater services; are resilient in the face of floods, drought, and other climate risks; have a role in decision-making processes related to water management in their communities; and share in the economic, social, and environmental benefits of water systems [69].” This is not the current experience of many residents in post-industrial cities, including many residents of Detroit, MI and across the U.S. Midwest. Sharing the direct words of residents, this qualitative study uniquely and urgently expresses the severity of recurrent household flood experiences and their potential environmental health effects in vulnerable communities. Leaders in post-industrial cities should consider if this is an underreported public health threat among their residents and seek to document the scale and severity, if so, perhaps through survey research or modeling. Resident narratives also provide practical recommendations for local public health, emergency preparedness, sustainability, water and sewage, and community leaders to work towards climate adaptation and improve risk communication targeting those most vulnerable economically and in health. Using plain language and locally relevant strategies and resources, risk communication messages for non-disaster flood events must be developed and tested while advancing theoretical frameworks. These narratives also suggest that flood-related local and federal relief programs must be assessed through additional research to prevent environmental health inequities, particularly in the context of disinvestment common to post-industrial regions. In inland urban areas, these experiences associated with frequent pluvial floods and related insights appear largely underreported by scholars and media except, perhaps, immediately following extreme and disaster-declared events. Although this paper largely focuses on climate adaptation, efforts towards mitigation are, of course, urgently needed to reduce future potential for extreme weather, including flood events.

## Figures and Tables

**Table 1:** Thematic codes and subcodes for data analysis.

Aging infrastructure	Economic factors or impacts
Disinvestment/bankruptcy	Lost living space (e.g., basement)
Stormwater management—community or neighborhood	Cultural or linguistic factors
Stormwater management—household level	Educational or literacy factors
Climate change	Pets
Clean up	Social support
Short term	Vulnerable populations
Long term	Health exposures/outcomes
Seeking assistance/support/relief	Mold
Non-response	Sewage
DTE Energy/utilities	Toxins
Landlords	Respiratory health
City	Infectious disease
Other (e.g., small business association, ngos)	Stress/trauma
Federal Emergency Management Agency	Safety/injury
Documentation of flooding (e.g., photos)	General quality of life
Description of household flooding (e.g., location, severity)	Health risk perceptions
Description of neighborhoods flooding	Transportation
Description of recurrence	Emergency preparedness
Appliances, heating & cooling systems	Media warnings/advisories
Electrical/power grid issues	Role of municipal leadership
Other orgs or agencies (e.g., United Way, Red Cross)	Additional feedback to community leaders
Insurance (e.g., housing, flood)	Comparisons to other geographies

**Table 2:** Demographic and household characteristics of study participants.

	Frequency (%)	Mean (SD)	Range
Age (years)		46.5 (17.2)	21–80
Gender (% Female)	9 (50.0)		
Race/ethnicity			
Black/African American	13 (72.2)		
Arab American	2 (11.1)		
Hispanic	2 (11.1)		
White	2 (11.1)		
Living in Detroit (years)	15 (83.3)	46.6 (17.1)	21–80
Living in Current Residence (years)		25.2 (18.0)	2–70
Owns home (vs. rents)			
Household flood events since 2014		4.2 (2.2)	3–10

**Table 3:** Examples of flooding-related health concerns reported by participants.

Theme	Sample Statements
Infectious disease	We have had incidents where we had rats in the city because people dump garbage in their neighborhoods and then floods come and it builds up ... that’s how your dogs get fleas and rabies and stuff. (District 1) We had a Hepatitis A * incident with two of our residents on the lower east side of the neighborhood, so I think that it’s—that’s a big concern for me. (District 3)
Mold	Also, black mold from after the flood goes away. And, you know, we live in homes that are over 100 years old, and you know, that’s brick and mortar. That space is destroyed. Like my girlfriend, their basement was finished and they had to get rid of all their drywall and all their everything because it had black mold in it. It affects your health very much. (District 5) Now when I called about the mold problem they told me, “Well, you can have someone come out.” But that’s gonna cost us money too, for them just to look at it and I just didn’t happen to have that kinda cash. So, I just had maybe a friend of a friend come look over it, and they told me it looked like something topical, and I could just go to home depot or something to look at it...but I don’t know ... to me it looked like it’s just, it didn’t looked like something I could just spray and get rid of. I keep spraying it and tried to get rid of it but there is certain parts of my basement that just the end part of the corner of my floor looks like black mold. (District 7)
Sewage	It’s not like a complete system that washes out constantly. It’s a system that builds up, releases, builds up, releases, and unfortunately during floods there is a lot of backup whether it’s through a drain or even through the houses personal unit. We have drains in the basement that are connected directly to sewage so if there’s that then and the sewage do get flooded they do get water in our house. And when we do get a flood we always mop it and wash it, you know, we use heavy detergent to try to clean which in itself is a health risk. (District 1) The smell was absolutely noxious and having to wait about 12 to 48 h to have that cleaned and some of the residents have done that cleaning on their own. (District 3)
Mental health	So I used to enjoy hearing the sound of rain, it was very calming, relaxing, and helped put me to sleep. Now it’s the source of great anxiety. It rained yesterday and the day before and the first thing I do is look in my basement, or if it’s pooling in the streets, because if it’s pooling in the streets then the system is not acting right and potentially could be a problem. (District 4)
Quality of life	I could probably have fully evaluated whether or not the three incidences I had in 2016, if they caused any personal health impacts, but I could imagine probably more extreme health-related issues like cancers or prolonged exposure to mold, headaches, chest aches, just body aches and they don’t know where these things are coming from and likely due to some allergen roaming in their house that is a result of some of type of sewer backup in the house. Those are some of the things that come to mind that people I know who’ve experienced basement floods, some of the physical ailments they’ve experienced. I noticed that, quite frankly. now that I’ve been thinking about it, that I’ve had far more headaches in the past couple years and I’ve never been a headache person. And I’ve never really considered that this could be a reason, I’d just take some Advil and keep it moving. (District 4)
Respiratory health	I have asthma and for me it was very challenging, and it was also a very hot day, and we have central airing in our house. So you have to keep the central air off because it will continue to have the sewage floating through the house. So it was hot and humid and sewage. I can’t describe what hot poop smells like but it was a really horrible smell. And for someone with asthma it was pretty bad. (District 3) So my brother gets pneumonia very easily. He had pneumonia very badly before...then he got pneumonia again. He was coughing, walking with pneumonia two weeks after [the flooding]. (District 5) Well, it got really bad. My allergies went crazy, and my eyes was watering, and I was just in bad shape there for a while. It just really affects me. That’s why I’m in such a hurry to clean it up, it’s because I know that if I don’t, that it will really affect me. (District 7)
Safety/injury	The generators we use cause carbon monoxide and that one time we did have that power outage from the floods for a week you could smell the carbon monoxide in the house so I’m sure that has had an effect on us. (District 1) Because where my bedroom is is where the furnace is beneath it so it was seeping carbon monoxide throughout the home. And it was a very scary experience, but the crazy thing is was that after DTE said that carbon monoxide was highest in this room. When they opened up the windows and turned off the furnace, and it was a cold snowy day. I remember it was a snowy night going into the next morning. I woke up the next morning with no headache no longer nauseous, no longer dizzy. I felt perfectly fine. I felt completely normal, and since then we’ve had the furnace replaced and I’ve been completely fine. (District 3) And at that church there was a story of an elderly woman who didn’t know what was happening in her basement, she thought it was just running water so she said let me go down and turn it off. And at that moment she slipped and fell and actually ended up breaking a bone in her lower leg. And she was in a cast. But to think that she slipped and fell in raw sewage, sitting there in raw sewage in pain in the basement of her home, and that was very sad to hear that story of that woman being injured by it. (District 3)

* Metro Detroit has experienced recent Hepatitis A outbreaks with 172 cases in Detroit between 1 August 2016–22 August 2018 [47]. No report or study has confirmed any of Detroit’s cases are associated with flood- or sewage-related exposures.

**Table 4:** Examples of flooding-related economic losses and costs as stressors reported by participants.

Theme	Sample Statements
Appliances	I can’t afford another flood. I would die. Literally, I’ll come back to life but I’ll die for about two seconds. It’s 3000 dollars, to know I already took one out [furnace] because the flood already damaged, and to put one in before I can fire it up for the winter? (District 3) I need the furnace, and I need the water tank, but everything is just, I can’t afford it now … so it still hanging on a thin thread. I did spend quite a bit. I just don’t have it in front of me right now. (District 4) A lot of loss. I mean, my last appliance died last week. A deep freezer full of food. And I was just like—at this point—I was just beyond the anger point. I mean, I’ve replaced two water heaters. I’ve replaced the washer and dryer. I’ve had numerous furnace issues, and all of those things just outright replace them. There’s no grant. That’s my own income coming to be diverted from other things to replace them because you have to have them. Just the toil of going through things that have been exposed to sewage and having to discard it, move it from a basement … I would probably say at this point in the tens of thousands. There are so many unaccounted for costs. I really don’t know what’s happening in my basement, and I’m concerned that I’m going to have to move considering how many floods I have and so close together, considering that the costs have been insurmountable. (District 4)
Damage assessment & clean up	But the cleanup in 2016—because of how noxious it was—we actually called a company to come sanitize and drywall. And that cost well over 1000 dollars, and I live in a home with my mother, who a single mother, and I am a student so that definitely was a big deal for us to pay for. (District 3) ... because to have somebody come out and look at it cost over a thousand dollars. I think that’s kinda extreme, that’s just somebody looking at it, not you know, (laughs) fixing the problem or even saying you know this is what you can get from just looking at it, this is mold and what type of mold. (District 7)
Housing	... a friend of mine who lost her Section 8 voucher because she couldn’t get her basement to stop flooding. As a result, she was homeless. (District 3)
Personal items	We have a senior population who lived in the neighborhood for a number of years. Some of them have raised their children and their grandchildren are there. So for people who have lived there for decades they have a lot of things in their basement. So for them it would be to have someone there to come and be able to do some that heavy lifting because some of that stuff is quite heavy. (District 3) We were in debt for a while too because of it. A lot of things that we had to replace. My mom had to get things from the thrift shop. She had to like rebuild it. (District 5)
Preventative strategies	Oh yeah, that [sump] pump was very expensive itself. The pump itself, I think it was over 1000 dollars and they had to come and actually dig through the cement in the basement and put the pipe down and also, the thing is, the pipe is not 100 percent either. Any company is like—this should work, but it’s not 100% guaranteed as well. So that’s a big concern: that it’s not a guarantee for us. (District 3)

**Table 5:** Examples of comments and suggestions for local municipal leaders reported by participants.

Theme	Sample Statements
Emergency planning	I wanna say a lack of, like there’s not many police officers and emergency that they can come to homes as quickly as they should … I mean we need a lot more emergency personnel. We need more police officers. We really do. We need that resource in our community. Now the bad part is it takes money ... a few more firemen, emergency officers, I think it would help us. I do. (District 6) ... more of just checking up. More of organizations, seeing if they need help. I know Americorps does a phenomenal security thing. CERT [Community Emergency Response Team] is amazing. In cases like that [the 2014 flood] where the fire trucks couldn’t get through, the people from CERT could communicate back and forth...what street they’re on. Even just the communication, for someone to be on the phone for them, and at least have available some communication network during this situation. (District 7) I look at it as more planning. I mean if there is an event, some of the nursing homes or some of the people who do live alone, if they are disabled or elderly, they need some type of planning. ‘Like, okay, I’m by myself. If there’s possibly a flood, possibly a snowstorm coming, is anybody here going to assist me or help me?’ I don’t know if it can be done but I look at it as a plan. What can they do in future planning? (District 7)
Other municipal services	We pay $300 on our tax bill for trash pick up. It’d be nice if when you have a flood event in your home and things need to be disposed of you can just haul them out to the curb and request a special pick up rather than that stuff having to sit out there until the next cycle, which is every two weeks. So if it floods on an off-week that means that’s going to be sitting on the curb which is a public health issue. It’s stuff that has been exposed to raw sewage …. (District 4)
Outreach & communication	Community leaders and healthcare providers need to advertise. We have this general feeling that everyone knows, but everyone doesn’t know. We should make sure everyone’s on the same page and that we do our best, you know, to have a strong standard for resources and information available to everyone in a variety of ways because otherwise people are missing out. And this is how residents and the City of Detroit find themselves in the situations that they’re in—from missing opportunities that they could have known about. (District 1)
Partnerships	With as many people as had moved in and out of the neighborhood. I didn’t know a lot of them and they didn’t know about such a thing as a community organization. They need to know that these organizations are in place so they can start asking questions. Community organizations should work directly with the City Council. (District 7)
Prevention & relief programs	I do wish that there were prevention programs, grants that would be used to address these issues, whether it’s putting a liner in your foundation for people who have basements making sure that they’re insulated and protected from standing water...I wish there were grants that would say if you have constant flooding or water or a lot of backup sewage there should be grants to help address it, and there’s not. (District 1) There should be a relief fund that somebody’s putting up for. Because we know that somebody is going to be tapping into this fund. We should not, as a municipality, be relying on FEMA, any federal anything. Locally, we know what we’re up against. It’s unnatural the way they built the whole city … so let’s stop acting like it’s a federal emergency when each city can contain some responsibility for what they’re doing. (District 3)
Stormwater infrastructure	And the other thing is that the water department should have some accountability. If you know these drains are bad and old you should replace them. You don’t mind us charging for drainage fees but you won’t correct the problem. (District 5)
Utilities	Utilities need to be publicly owned. And, while the Great Lakes Water Authority is a publicly owned utility, it has moved towards privatization and the basis for service shouldn’t be to satisfy bond holders, it should be to satisfy the people. (District 5)
